# Optimization of spray operation parameters of unmanned aerial vehicle confers adequate levels of control of fall armyworm (*Spodoptera frugiperda*)

**DOI:** 10.3389/fpls.2025.1581367

**Published:** 2025-05-08

**Authors:** Ying Liu, Xiao Liang, Chunling Wu, Xingkui An, Mufeng Wu, Zihua Zhao, Zhihong Li, Qing Chen

**Affiliations:** ^1^ Environment and Plant Protection Institute, Chinese Academy of Tropical Agricultural Sciences/Key Laboratory of Integrated Pest Management on Tropical Crops, Ministry of Agriculture and Rural Affairs, Haikou, China; ^2^ Sanya Research Academy, Chinese Academy of Tropical Agriculture Science/Hainan Key Laboratory for Biosafety Monitoring and Molecular Breeding in Off-Season Reproduction Regions, Sanya, China; ^3^ Sanya Institute of China Agricultural University, Sanya, China

**Keywords:** unmanned aerial vehicle, fall armyworm, spray operation parameter, droplet deposition, control effect

## Abstract

**Introduction:**

The fall armyworm (FAW), *Spodoptera frugiperda*, is a serious threat to maize production. Unmanned aerial vehicles (UAVs) represent a promising method for controlling FAW outbreaks. Given that FAW larvae primarily feed inside the maize whorl, we hypothesized that the efficacy of insecticide application depends on droplet density and coverage rate on the upper maize canopy.

**Methods:**

This study evaluated the effects of spray operation parameters—including three flight heights (2.0, 2.5, and 3.0 m), three spray volumes (30.0, 37.5, and 45.0 L ha^-1^), and two nozzle types (XR11001VS and XR110015VS)—on droplet deposition distribution in maize canopies. Additionally, the control efficacy of 35% chlorantraniliprole water dispersible granules (WDG) against FAW was assessed over three consecutive years (2019-2021) to determine its correlation with droplet deposition.

**Results:**

Results indicated that flight height, spray volume, and nozzle type significantly influenced droplet deposition distribution. Two treatments—XR110015VS nozzle with 37.5 L ha^-1^ spray volume at 2.5 m flight height and XR110015VS nozzle with 45.0 L ha^-1^ spray volume at 2.5 m flight height—achieved the highest droplet density, optimal coverage rate on the upper maize canopy, and the lowest damage index, confirming our hypothesis. These treatments also demonstrated comparable FAW control efficacy to traditional electric air-pressure knapsack sprayers.

**Discussion:**

The findings provide practical insights for optimizing UAV-based insecticide applications to improve FAW management in maize production systems.

## 1 Introduction

Maize (*Zea mays* L.), cultivated across 44.22 million hectares in China (National Bureau of Statistics of China, 2024), faces a significant threat from the Fall armyworm (FAW), *Spodoptera frugiperda* (J. E. Smith, 1797) (Lepidoptera: Noctuidae). Originating in the Americas (Goergen et al., 2016; Bateman et al., 2018; Boaventura et al., 2020), FAW has spread to 22 Chinese provinces (Shan et al., 2022) driven by its adaptability to diverse hosts and environments (Nagoshi et al., 2019; Chinwada et al., 2023), high reproductive capacity (Bateman et al., 2018; Kumar et al., 2022), and long-distance migratory ability (Nagoshi et al., 2012; Westbrook et al., 2016). Early-instar larvae typically hide in the maize whorl and feed on upper leaves, creating small window panes (Bateman et al., 2018), while late-instar larvae bore into maize cobs to feed on kernels (Kansiime et al., 2019), making control challenging.

Traditionally, chemical insecticides applied using knapsack sprayers have been the primary FAW control method in China (Ren et al., 2019; Wang G. et al., 2019). However, these methods are inefficient and pose significant risks to the environment and human health, especially during critical growth stages such as the late whorl and tasseling stages. Therefore, improving the mechanization and precision of pesticide applications is essential for effective FAW control while minimizing environmental and operator risks.

Unmanned Aerial Vehicles (UAVs) have emerged as a promising alternative, offering enhanced mobility, reduced labor costs, and lower operator exposure compared to manual or ground-based spraying (Wang G. et al., 2018; Zhang et al., 2018). UAVs have a working efficiency of 4–10 hectares per hour, which is 30 to 100 times higher than manual spraying and 4 to 33 times higher than ground-based sprayers (Yang et al., 2018). This high efficiency makes UAVs particularly suitable for covering large areas within narrow optimal application windows, crucial for effective FAW control.

The effectiveness of UAV-based pesticide application depends significantly on key operational parameters such as spray volume, nozzle type, and flight height, which influence droplet deposition and control efficacy. For example, higher spray volumes and coarser nozzles improve deposition and control efficacy for wheat aphids and powdery mildew (Wang G. et al., 2019), while larger droplet sizes enhance penetration and uniformity in rice canopies (Chen et al., 2020). Similarly, higher flight heights increase droplet coverage and deposition in cotton (Lou et al., 2018). Taking into account the specific infestation patterns and behaviors of pests is also crucial when applying insecticides via UAV. Qin et al. (2016) obtained a better control efficacy against the brown planthopper by optimizing operation height and velocity, with the highest droplet deposition at a spraying height of 1.5 m and a speed of 3 m·s^-1^ on the lower canopy of rice, where planthoppers generally occur. Given that FAW larvae primarily feed in the maize whorl, we hypothesize that optimizing UAV spray parameters to maximize droplet deposition on the upper canopy will significantly enhance FAW control.

This study systematically evaluates droplet deposition and distribution on maize canopies under varying UAV parameters, including spray volume, nozzle type, and flight height. By optimizing these parameters, we aim to maximize deposition on the maize whorl, improving FAW control. The findings will guide aerial applications of chlorantraniliprole and support sustainable pest management in maize cultivation.

## 2 Materials and methods

### 2.1 Insecticide, maize and field site

The insecticide used in this study was 35% chlorantraniliprole Water Dispersible Granules (WDG) applied at a dose of 52.5 g active ingredient (a.i.) per hectare (Bayer Crop Science Co., Ltd., Beijing, China). The maize cultivar selected for the experiment was ‘Meilan Huangguan’ (Hainan Lvchuan Seed Co., Ltd., Haikou, China). The experimental field was arranged with row spacing of approximately 65 cm, plant spacing of 35 cm, and an average plant height of 65 cm during the application period. The study was conducted at the Chinese Academy of Tropical Agricultural Sciences in Danzhou, Hainan Province, China. This location was chosen due to its favorable climate conditions for maize cultivation and its history of FAW infestations, making it an ideal setting for evaluating the effectiveness of UAV-based pesticide applications.

### 2.2 Record of climatic conditions

Weather parameters, including wind speed, were monitored and recorded using the Smart Sensor AR866A Anemometer (Dongguan Wanchuang Electronic Products Co., Ltd., Dongguan, China). Air temperature and relative humidity were measured with the Smart Sensor AR837 Temperature and Humidity Gauge (Dongguan Wanchuang Electronic Products Co., Ltd., Dongguan, China). The air temperature ranged from 24.2°C to 35.5°C, while the relative humidity varied between 46.2% and 54.6%. Wind speeds in all test treatments remained below 1.5 m/s. Detailed weather data for each test treatment are provided in **Supplementary Table S1**.

### 2.3 Spraying platform and spraying systems

**Figure 1:**
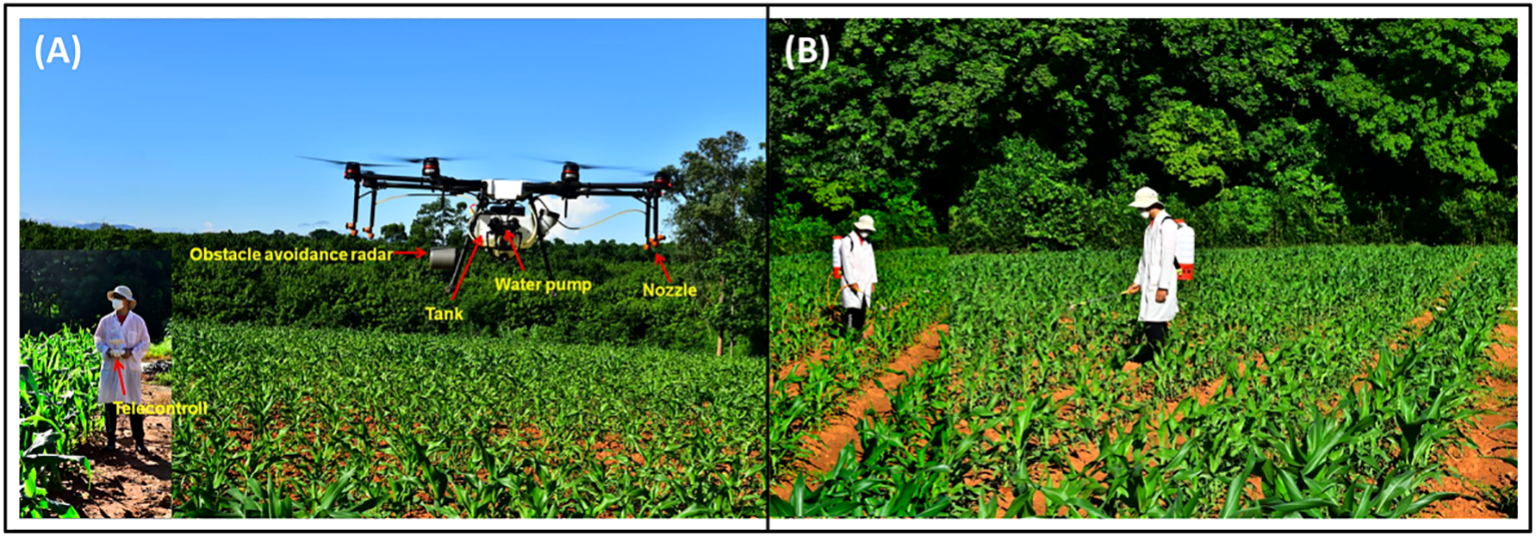
The DJI MG-1P UAV sprayer and the operator conducting the spray operation **(A)**; electric air-pressure knapsack (EAP) sprayer (SX-MD18DA) used in the experiment **(B)**.

The eight-rotor agricultural UAV (MG-1P, SZ DJI Technology Co., Ltd., Shenzhen, China) employed in this study featured a modular spraying system comprising a 10 L chemical tank, dual customized diaphragm pumps, four vertically mounted flat-fan nozzles, and an integrated radar altimeter for real-time altitude adjustment and obstacle avoidance (**Figure 1A**). Nozzles were symmetrically affixed to four spray booms aligned with the UAV arms, oriented vertically downward to optimize droplet trajectory within the rotor-induced airflow. An intelligent planning operation mode was utilized, allowing dynamic adjustments to flow rate, spraying width, spray volume, and flight speed based on site-specific conditions. For comparative analysis, a commercial electric air-pressure (EAP) knapsack sprayer (SX-MD18DA, Zhejiang Xixia Sprayer Co., Ltd., Taizhou, China) was deployed (**Figure 1B**).

### 2.4 Experimental design

**Figure 2:**
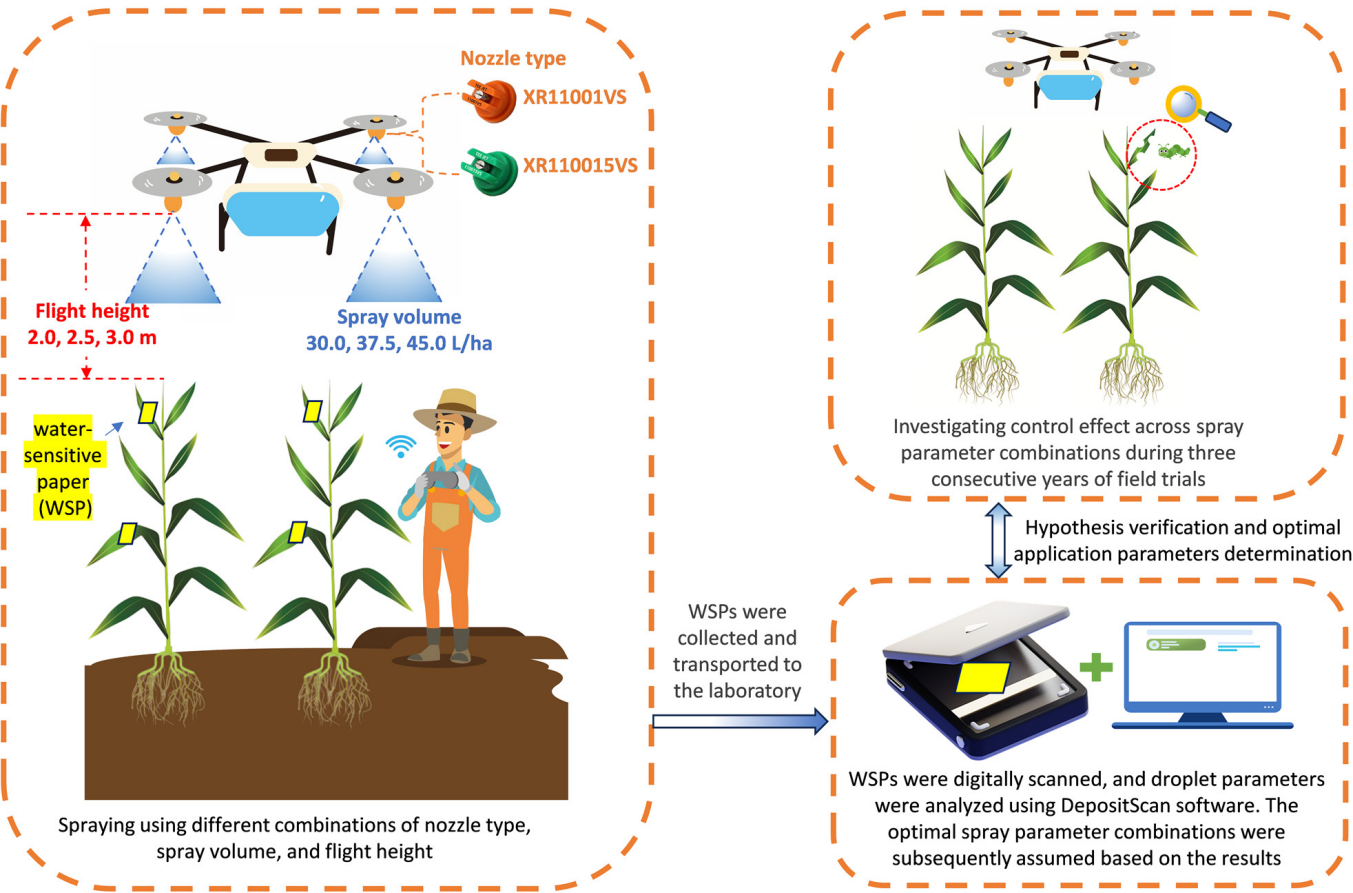
Technical workflow of the study.

In 2019, a study was conducted to investigate the influence of various spray operation parameter combinations (i.e., nozzle type, flight height, and spray volume) on insecticide droplet deposition on maize canopies and the control efficacy against FAW. The objective was to determine the optimal spray operation parameters. From 2020 to 2021, two consecutive years of field trials were carried out to further validate the relationship between control efficiency and droplet deposition distribution. Maize sowing dates were June 15, 2019; August 15, 2020; and July 25, 2021. Throughout these experiments, maize was consistently at the whorl stage of growth. **Figure 2** illustrated the technical workflow of this study.

### 2.5 Treatments and spray operation parameters

**Figure 3:**
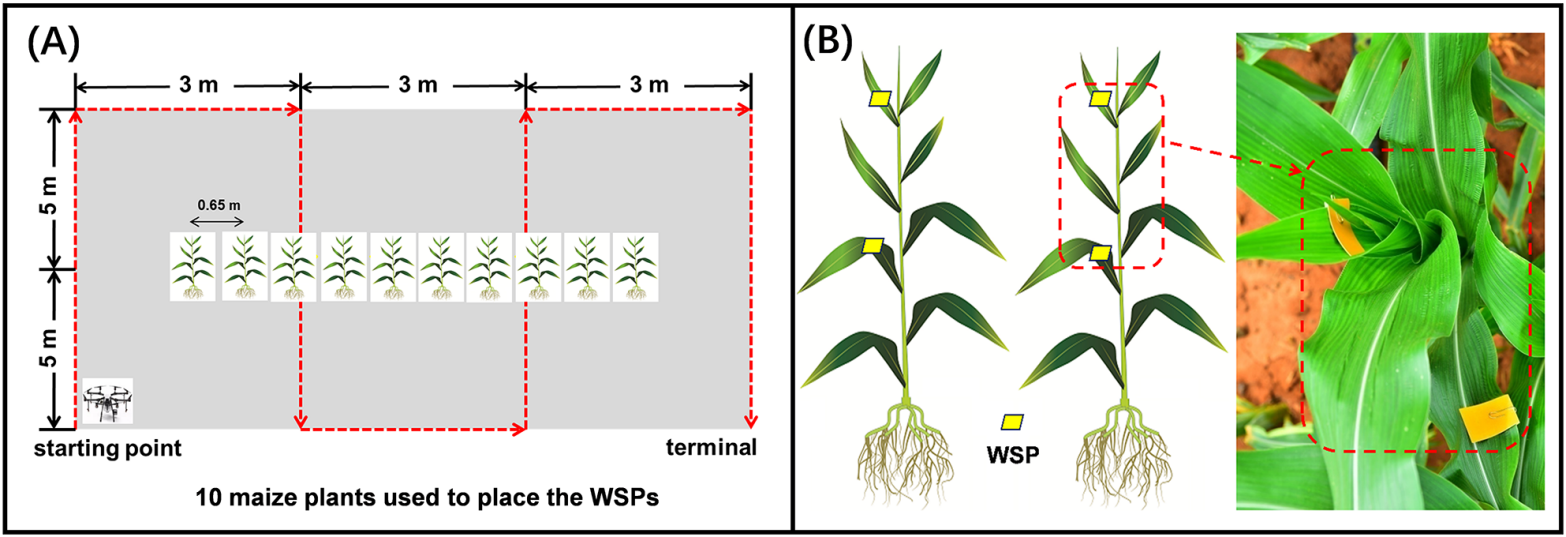
Schematic diagram of the site layout and flight route **(A)**; placement of WSPs at each sampling position within the maize canopy **(B)**.


Twenty treatments were employed in this study, including eighteen UAV treatments, one EAP treatment and an untreated control. The spray operation parameters for each treatment are summarized in **Table 1**, with each treatment replicated three times. The experimental design followed a randomized block design, as detailed in **Supplementary Table S2**. Each replicate consisted of a 10 m × 9 m (90 m^2^) plot of maize arranged in a rectangular shape (**Figure 3A**). To minimize drift pollution, treatment plots were separated by a 10 m buffer zone. For the UAV treatments, we evaluated the influence of flight height (2.0, 2.5, and 3.0 m), nozzle type (XR11001VS and XR110015VS), and spray volume (30.0, 37.5, or 45.0 L·ha^-1^) on droplet density, coverage rate, and droplet size across both the upper and lower canopies of maize plants. The UAV flight route is illustrated in **Figure 3A**. The red arrow indicates the flight route. The UAV turned around approximately 10 m from the starting point and completed four round trips before reaching the terminal point. After setting the spraying width, spray volume and nozzle discharge, the flight velocity was kept at an automatic setting. The spray volume, spraying width, nozzle discharge and flight velocity were converted using the following equation (Tang et al., 2018).

**Table 1:** Spray operation parameters in each treatment.

Treatment	Spraying equipment	Flight height (m)	Nozzle type	Spray volume (L·ha^-1^)	Travelling speed (m·s^-1^)	Spraying width (m)	Nozzle discharge (L·min^-1^)
1	UAV	2.0	XR11001VS	30.0	3.3	3	1.8
2				37.5	2.7	3	1.8
3				45.0	2.2	3	1.8
4			XR110015VS	30.0	4.6	3	2.5
5				37.5	3.7	3	2.5
6				45.0	3.1	3	2.5
7		2.5	XR11001VS	30.0	3.3	3	1.8
8				37.5	2.7	3	1.8
9				45.0	2.2	3	1.8
10			XR110015VS	30.0	4.6	3	2.5
11				37.5	3.7	3	2.5
12				45.0	3.1	3	2.5
13		3.0	XR11001VS	30.0	3.3	3	1.8
14				37.5	2.7	3	1.8
15				45.0	2.2	3	1.8
16			XR110015VS	30.0	4.6	3	2.5
17				37.5	3.7	3	2.5
18				45.0	3.1	3	2.5
19	EAP	-^a^	Hollow cone nozzle	450.0	0.3	-^b^	0.8
20	Control	–	–	–	–	–	–

a Nozzle moved from the top to the bottom of the canopy and the droplets distributed over the canopy layer as soon as possible.

b Sprayed along the maize row and the row spacing was 0.65 m.

$$d=\frac{\nu \times w\times V}{166.67}$$

where d is the nozzle discharge (L·min^-1^), V is the spray volume (L·ha^-1^), w is the spraying swath (m), and *v* is the flight velocity of the UAV (m·s^-1^). In this study, we selected two commonly used nozzles, TEEJET XR11001VS and TEEJET XR110015VS, for the DJI MG-1P sprayer. These nozzles were chosen due to their ability to accurately calibrate discharge when used interchangeably (Guo et al., 2020). The XR110015VS nozzle has a higher flow rate of 0.15 gallons per minute and larger droplet size of 202 μm compared to the XR11001VS nozzle, which has a flow rate of 0.10 gallons per minute and droplet size of 174 μm, both measured at a pressure of 0.276 MPa (Yu et al., 2020). Additionally, for the XR11001VS nozzle, the droplet diameter range is 130–250 μm, while for the XR110015VS nozzle, the range is 170–265 μm (available at https://www.dji.com/cn/products/compare-agriculture). In this study, the working pressure for both nozzles was set to 0.4 MPa. The UAV and EAP sprayer were operated by well-trained operators. The EAP application was conducted at a spray volume of 450 L·ha^-1^, a working pressure of 0.4 MPa, and a travelling speed of approximately 0.3 m/s.

### 2.6 Sample scheme and characterization of droplet parameters

In this study, water-sensitive paper (WSP, 30 mm×80 mm, Chongqing Liuliu Shanxia Plant Protection Technology Co., Ltd., Chongqing, China) was utilized to characterize droplet characteristics and canopy distribution. Prior to each application treatment, two WSPs were placed in the upper canopy (the seventh leaf, near the maize whorl) and the lower canopy (the third leaf) of the maize plants (**Figure 3B**). Two WSPs were placed on each of ten maize plants per plot, with each plant separated by 0.65 m (**Figure 3A**). The objectives of using the WSPs were to assess droplet parameters, including droplet density, coverage rate and droplet size at different canopy levels. Specifically, the WSP placed on the seventh leaf (located in the maize whorl) was crucial because the droplet deposition on this site can best represent the FAW control efficiency. After each spraying treatment, WSPs were collected and bagged in zip-lock plastic bags labeled with the treatment and replicate information. The WSPs were scanned as digital images at a resolution of 600 dpi. Droplet parameters (droplet density, coverage rate, and droplet size) were analyzed using the DepositScan software (Zhu et al., 2011). The analysis of droplet size followed the method described by Tang et al. (2018). The influence of operation parameters on droplet density, coverage rate and droplet size on both the upper and lower canopies of maize plants was analyzed for UAV treatments. However, droplet parameters for EAP treatments were not shown due to the excessively large spray volume, which precluded meaningful droplet distribution analysis on the WSP.

### 2.7 Control effect against FAW

The damage index was used to evaluate the control effect of each treatment against FAW. The damage index for each treatment plot was calculated according to the damage index equation.


$$Damage\;index=\frac{\sum \left(Number\;of\;damage\;leaves\;at\;each\;level\;\times \;Corresponding\;level\;value\right)}{Total\;number\;of\;investigation\;\times 9}\times 100$$

This evaluation was conducted on new leaves of 50 maize plants in each plot at 3 days and 7 days after treatment (DAT), using a modified scale of 0–9 developed by Davis et al (Toepfer et al., 2021). The specific field investigation method was as follows: a five-point sampling method (W-pattern approach) was employed in each plot, and at each point, the scout assessed 10 plants for signs of FAW feeding damage (refer to the damage index scale). During the initial assessment at 3 DAT, all maize plants were marked with red string to ensure consistent monitoring of the same plants at 7 DAT (Shan et al., 2022).

### 2.8 Statistical analysis

Data were analyzed using SPSS v. 19.0 (SPSS Inc., Chicago, IL, USA). Prior to analysis, data normality was assessed using the Shapiro-Wilk test (*P* > 0.05) and homogeneity of variance was evaluated using Levene’s test (*P* > 0.05). To stabilize variances and meet normality assumptions, the coverage rate was transformed using y = arcsin√X/100, while droplet density and size were log (X + 1) transformed. One-way ANOVA was used to detect significant differences in droplet parameters (droplet density, coverage rate, and droplet size) or damage index among treatments, followed by the Tukey’s *post hoc* test with *P*< 0.05. Student’s t-test was used to compare significant differences in droplet parameters between the two nozzle types.

## 3 Results

### 3.1 Effect of spray volume and nozzle type on droplet deposition characteristics in maize canopies at different flight heights

**Figure 4:**
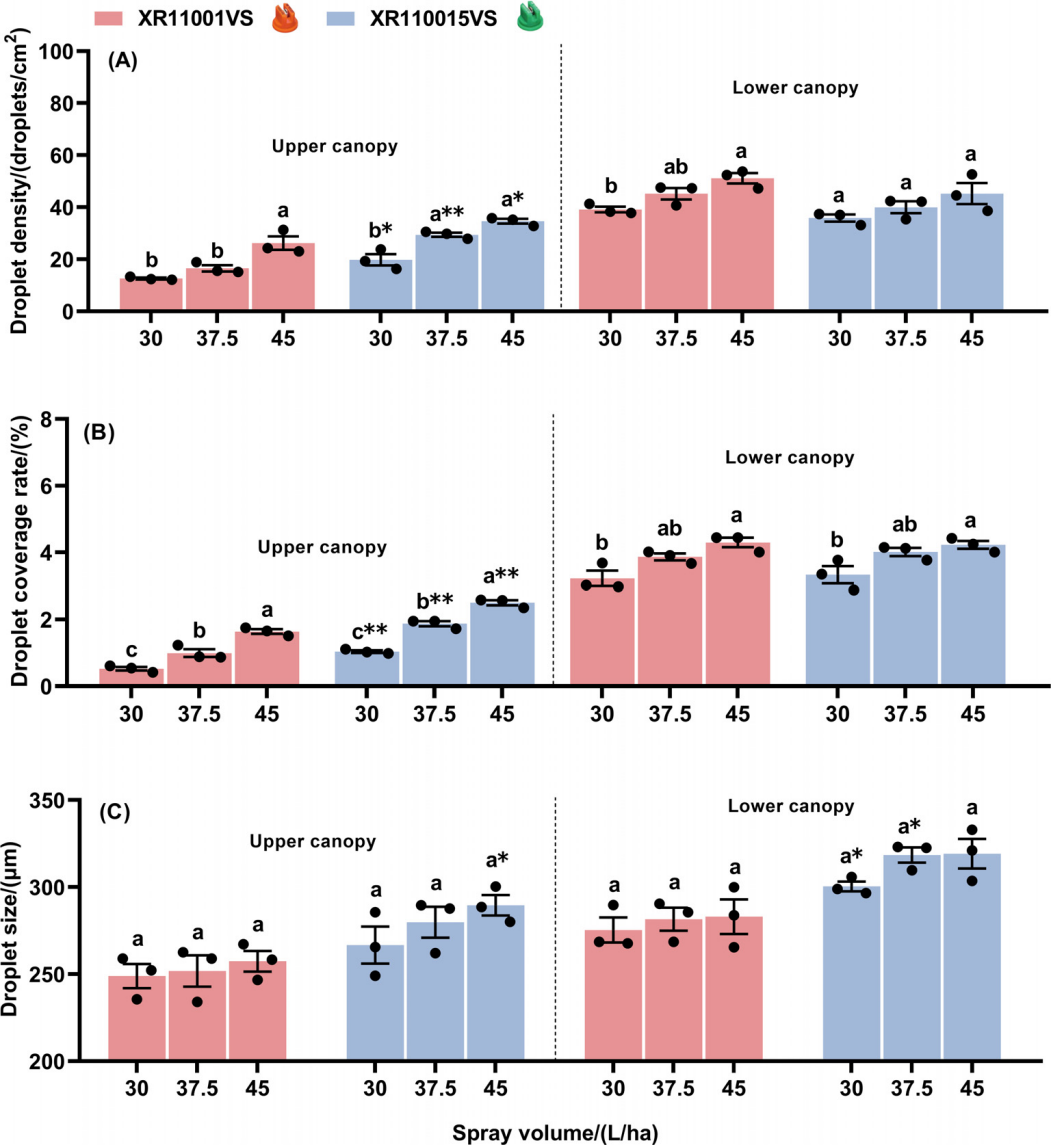
Droplet density **(A)**, droplet coverage rate **(B)** and droplet size **(C)** on the upper and lower canopies of maize plant with two nozzle types, across three spray volumes, at a fixed flight height of 2.0 m. Different letters on bars indicate significant differences (*P*< 0.05) among spray volumes. Asterisks above the bars denote significant difference of droplet density (droplet coverage rate or drop size) between two nozzles by using t-test, at a certain spray volume on a same canopy. * and ** indicate significant difference at the significance levels of 0.05 and 0.01, respectively; ns represents no significance. Error bars denote standard error of the means.

Effect of spray volume and nozzle type on droplet deposition characteristics in maize canopies at the flight height of 2.0 m are shown in **Figure 4**. At a flight height of 2.0 m using the nozzle XR11001VS, spray volume significantly influenced droplet density and coverage rate in both the upper and lower canopies (**Figures 4A, B**). Both droplet density and coverage rate generally increased with spray volume, with a significant increase observed when comparing the lowest (30 L/ha) and highest (45 L/ha) spray volumes. Droplet size remained consistent across different spray volumes for both the upper and lower canopies (**Figure 4C**). When using the nozzle XR110015VS, droplet density on the upper canopy was lowest at 30 L/ha and highest at 37.5 and 45 L/ha. No significant differences in droplet density were detected on the lower canopy. The coverage rate on the upper canopy increased significantly with spray volume. In contrast, on the lower canopy, the coverage rate at 37.5 L/ha did not differ significantly from either 30 or 45 L/ha. Droplet size did not vary significantly with changes in spray volume for both the upper or lower canopies. Furthermore, when comparing the effects of the two nozzle types (XR110015VS and XR11001VS) at different spray volumes, we found droplet density and coverage rate were significantly higher with the XR110015VS nozzle at all tested volumes. In contrast, no significant differences in droplet density or coverage rate were observed on the lower canopy between the two nozzle types. Regardless of canopy position, the droplet size produced by the nozzle XR110015VS was consistently larger than that of the nozzle XR11001VS at the same spray volume. Significant differences in droplet size were observed on the upper canopy at a spray volume of 45 L/ha and on the lower canopy at spray volumes of 30 L/ha and 37.5 L/ha. In summary, the XR110015VS nozzle demonstrated superior droplet density and coverage on maize whorls across three spray volumes at a 2.0 m flight height compared to the XR11001VS nozzle.

**Figure 5:**
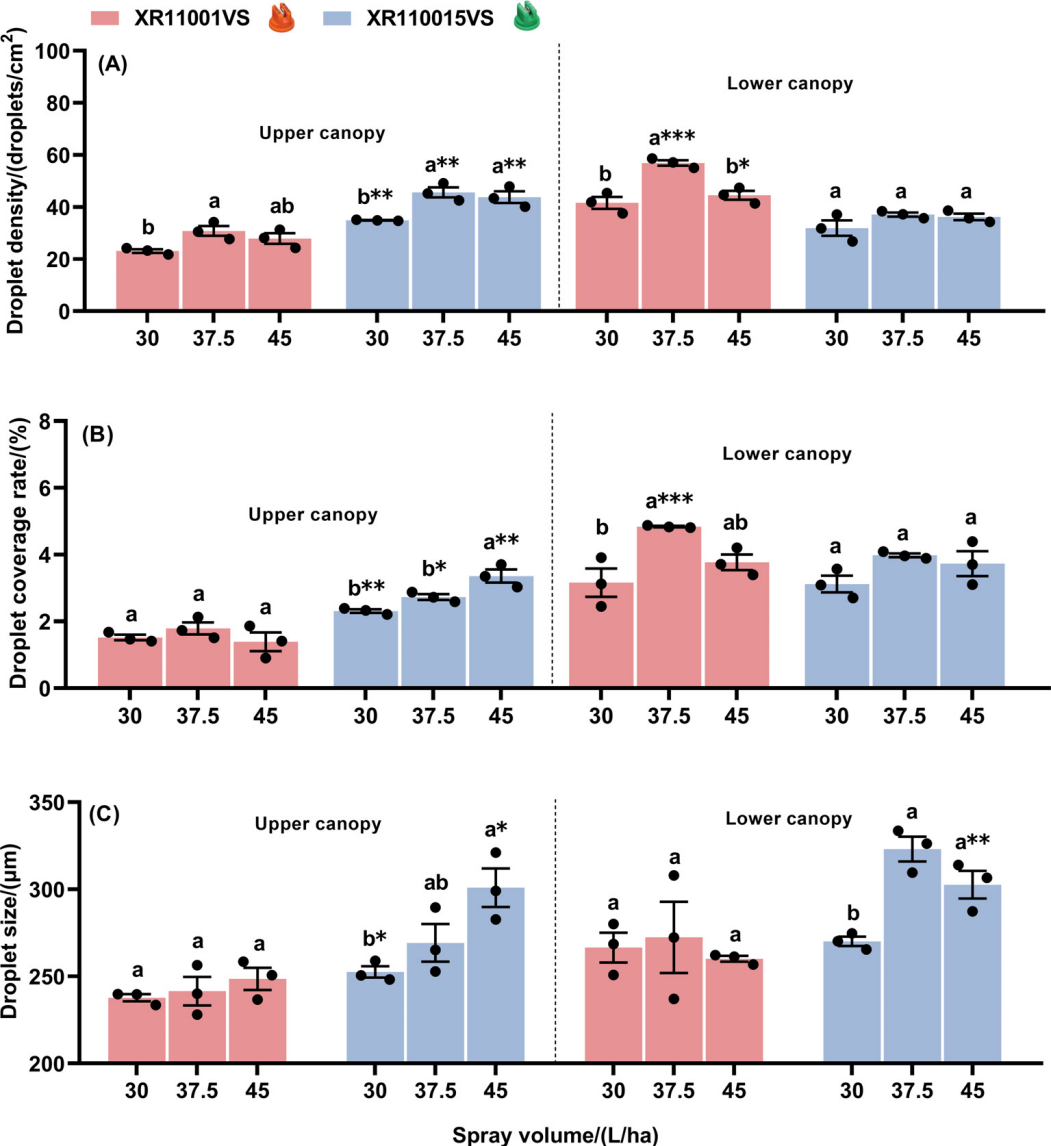
Droplet density **(A)**, droplet coverage rate **(B)** and droplet size **(C)** on the upper and lower canopies of maize plant with two nozzle types, across three spray volumes, at a flight height of 2.5 m. Different letters on bars indicate significant differences (*P*< 0.05) among spray volumes. Asterisks above the bars denote significant difference of droplet density (droplet coverage rate or drop size) between two nozzles by using *t*-test, at a certain spray volume on a same canopy. *, ** and *** indicate significant difference at the significance levels of 0.05, 0.01 and 0.001 levels, respectively; ns represents no significance. Error bars denote standard error of the means.

Effect of spray volume and nozzle type on droplet deposition characteristics in maize canopies at the flight height of 2.5 m are shown in **Figure 5**. At a height of 2.5 m using nozzle XR11001VS, spray volume significantly influenced droplet density on both the upper canopy and the lower canopy (**Figure 5A**). For droplet coverage rate, significant variation was observed among different spray volumes on the lower canopy, while no significant difference was detected on the upper canopy (**Figure 5B**). The highest droplet density and coverage rate were achieved with a spray volume of 37.5 L·ha^-1^ for both the upper and lower canopies. Additionally, droplet size did not show statistically significant differences among spray volumes on either the upper or lower canopy (**Figure 5C**). When using the nozzle XR110015VS, droplet densities at spray volumes of 37.5 and 45.0 L ha^−1^ were significantly higher than at 30.0 L ha^−1^ on the upper canopy, but no significant differences were observed on the lower canopy (**Figure 5A**). For droplet coverage rate, a volume of 45.0 L·ha^-1^ resulted in significantly higher coverage compared to 30.0 L·ha^-1^ and 37.5 L·ha^-1^ on the upper canopy, whereas no significant difference was found on the lower canopy (**Figure 5B**). Spray volume significantly influenced droplet size on both the upper canopy and the lower canopy (**Figure 5C**). On the upper canopy, droplet size generally increased with spray volume, while on the lower canopy, droplet size at spray volumes of 37.5 and 45.0 L ha^−1^ were significantly larger than at 30.0 L ha^−1^. Furthermore, when comparing the two nozzles (XR110015VS and XR11001VS), we found significant differences in performance. For the upper canopy, the XR110015VS nozzle exhibited significantly higher droplet density and coverage rate at all tested volumes. In contrast, for the lower canopy, the XR11001VS nozzle performed better. The droplet density using the XR11001VS nozzle was significantly higher than that of the XR110015VS nozzle at spray volumes of 37.5 L·ha^-1^ and 45.0 L·ha^-1^. The coverage rate using the XR11001VS nozzle was significantly higher at 37.5 L·ha^-1^. Regardless of whether on the upper or lower maize canopy, the droplet size produced by the nozzle XR110015VS was consistently larger than that of the nozzle XR11001VS at the same spray volume. Significant differences were observed on the upper canopy at spray volumes of 30 L/ha and 45.0 L/ha, and on the lower canopy at a spray volume of 45.0 L/ha. In summary, the XR110015VS nozzle demonstrated superior droplet density and coverage on maize whorls across three spray volumes at a 2.5 m flight height compared to the XR11001VS nozzle.

**Figure 6:**
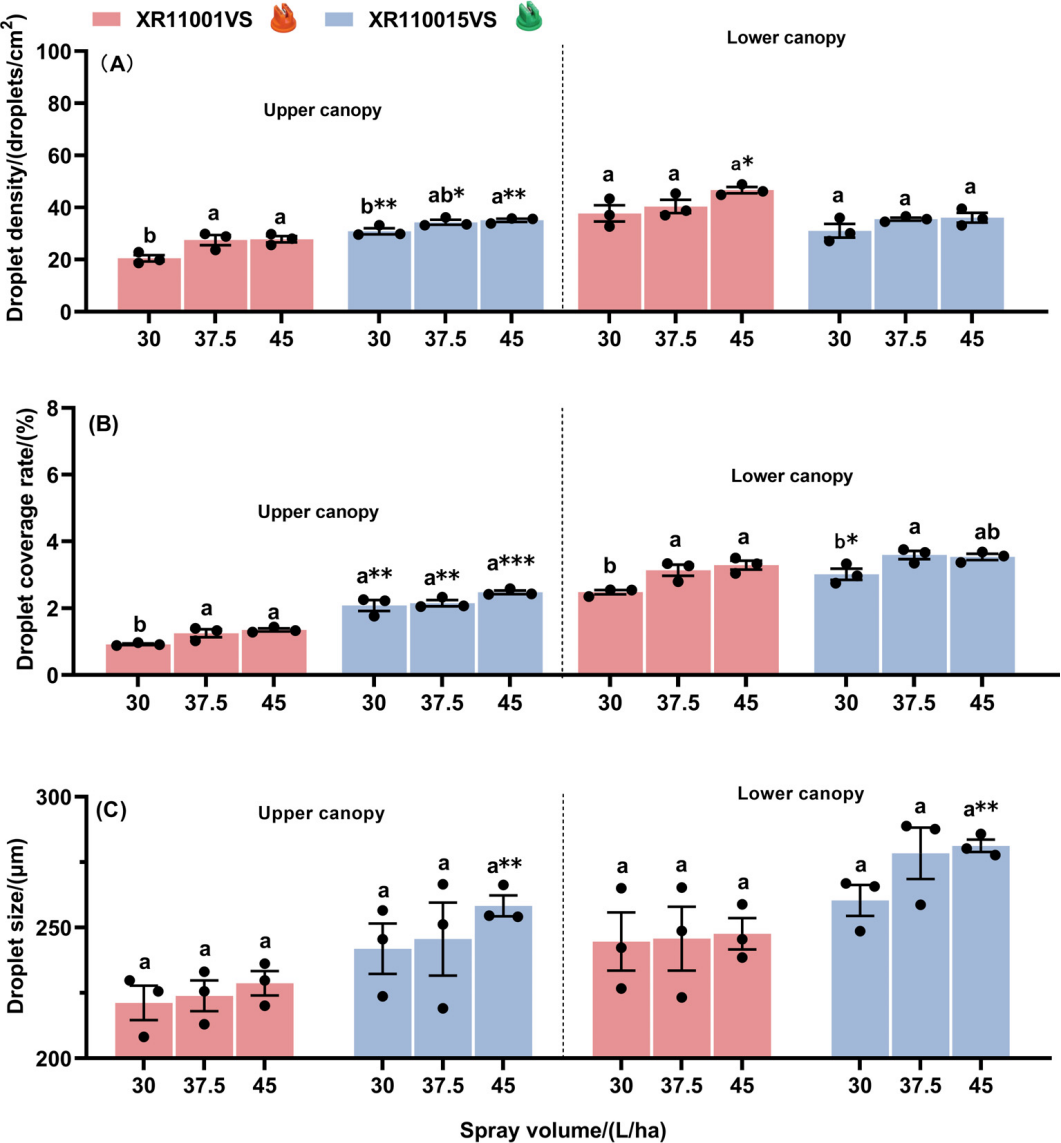
Droplet density **(A)**, droplet coverage rate **(B)** and droplet size **(C)** on the upper and lower canopies of maize plant with two nozzle types, across three spray volumes, at a flight height of 3.0 m. Different letters on bars indicate significant differences (*P*< 0.05) among spray volumes. Asterisks above the bars denote significant difference of droplet density (droplet coverage rate or drop size) between two nozzles by using *t*-test, at a certain spray volume on a same canopy. *, ** and *** indicate significant difference at the significance levels of 0.05, 0.01 and 0.001 levels, respectively; ns represents no significance. Error bars denote standard error of the means.

Effect of spray volume and nozzle type on droplet deposition characteristics in maize canopies at the flight height of 3.0 m are shown in **Figure 6**. At a flight height of 3.0 m using the nozzle XR11001VS, spray volume significantly influenced droplet density on the upper canopy, but no significant difference was detected on the lower canopy (**Figure 6A**). Droplet coverage was lowest at 30 L/ha and highest at both 37.5 and 45 L/ha in both the upper and lower canopies (**Figure 6B**). Both droplet density and coverage rate demonstrated a progressive increase with higher spray volumes. Droplet size remained unaffected by spray volume on both the upper and the lower canopy (**Figure 6C**). When using the nozzle XR110015VS, spray volume significantly influenced droplet density on the upper canopy, whereas no significant difference was detected on the lower canopy (**Figure 6A**). For droplet coverage rate, no significant differences were detected on the upper canopy, while spray volume significantly influenced coverage rate on the lower canopy (**Figure 6B**). Both droplet density and coverage rate demonstrated a progressive increase with higher spray volumes. The droplet size did not vary with spray volume on either the upper canopy or the lower canopy (**Figure 6C**). Furthermore, when comparing the two nozzles (XR110015VS and XR11001VS), we found that for the upper canopy, the XR110015VS nozzle exhibited significantly higher droplet density and coverage rate at all tested volumes. For the lower canopy, droplet density and coverage rate showed no significant differences between the two nozzle types except for two specific cases. The droplet density using the XR11001VS nozzle was significantly higher than that of the XR110015VS nozzle at a spray volume of 45 L·ha^-1^. Additionally, the coverage rate using the XR110015VS nozzle was significantly higher than that of the XR11001VS nozzle at a spray volume of 37.5 L·ha^-1^. Regardless of whether on the upper or lower maize canopy, the droplet size produced by the nozzle XR110015VS was consistently larger than that of the nozzle XR11001VS at the same spray volume. Significant differences in droplet size were observed at a spray volume of 45.0 L/ha both on the the upper canopy and the lower canopy. In summary, the XR110015VS nozzle demonstrated superior droplet density and coverage on maize whorls across three spray volumes at a 3.0 m flight height compared to the XR11001VS nozzle.

To sum up, the XR110015VS nozzle demonstrated superior droplet density and coverage rates on maize upper canopies compared to the XR11001VS nozzle. Given these findings, we selected the nozzle XR110015VS for further analysis.

### 3.2 Determination of optimal flight height and spray volume

**Figure 7:**
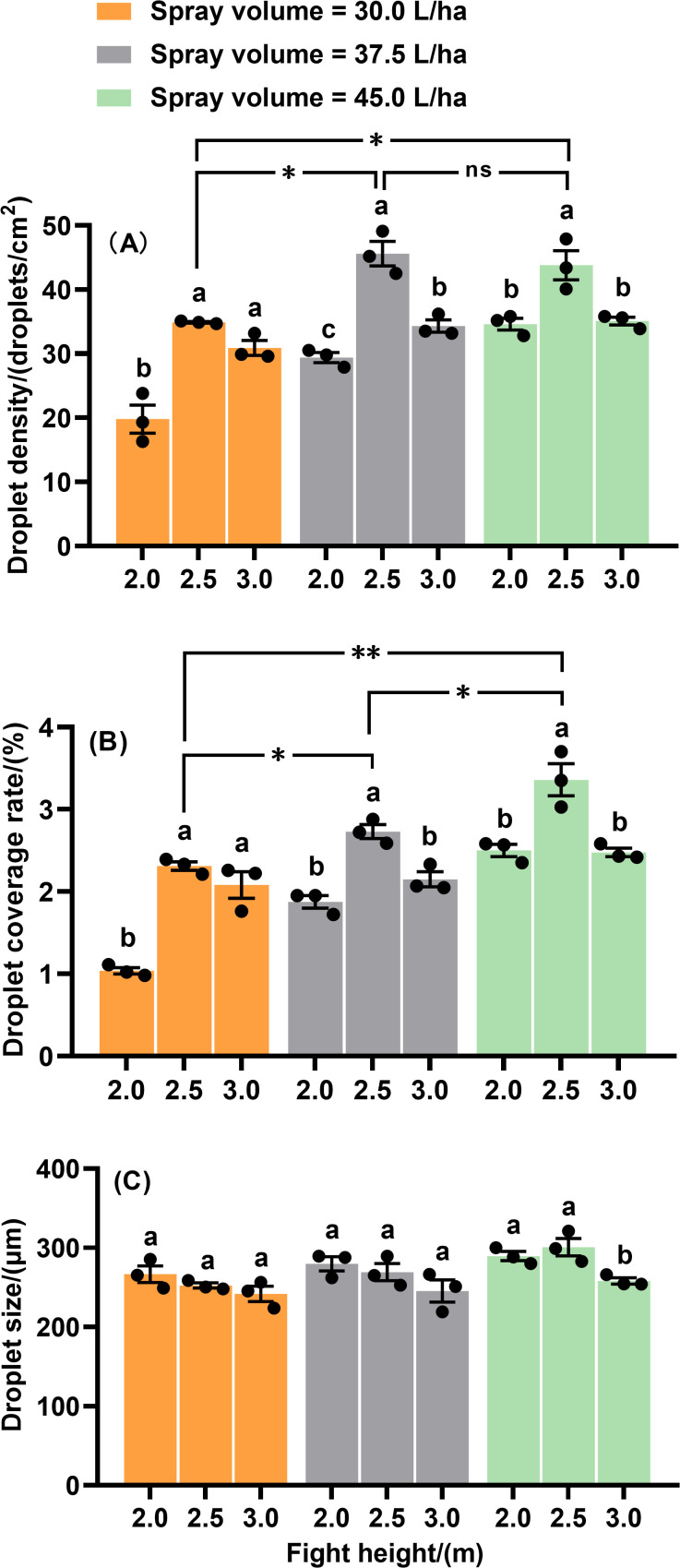
Droplet density **(A)**, droplet coverage rate **(B)** and droplet size **(C)** on the upper canopy of maize plant with nozzle XR110015VS at three flight heights. Different letters on bars indicate significant differences (*P*< 0.05) among flight heights. Asterisks above the bars denote significant difference of droplet density (droplet coverage rate) between spray volumes at the flight height of 2.5 m by using *t*-test. * and ** indicate significant difference at the significance levels of 0.05 and 0.01, respectively; ns represents no significance. Error bars denote standard error of the means.

When using the XR110015VS nozzle, the effects of flight height on droplet deposition characteristics on the upper canopy were systematically compared and analyzed across different spray volumes (**Figure 7**). Flight height had significant effects on droplet density and coverage rate at all tested spray volumes on the upper canopy. The highest droplet density and coverage rate were observed at a flight height of 2.5 m (**Figures 7A, B**). Additionally, at a flight height of 2.5 m, the spray volumes of 37.5 L·ha^-1^ and 45.0 L·ha^-1^ resulted in higher droplet density and coverage rate compared to 30.0 L·ha^-1^ (**Figures 7A, B**). For droplet size, only at a spray volume of 45.0 L·ha^-1^ did flight height show a significant effect. Specifically, droplet sizes at flight heights of 2.0 m and 2.5 m were significantly larger than those at 3.0 m (**Figure 7C**). Based on the above results, we assume that the optimal spray operation parameter combinations are a flight height of 2.5 m combined with spray volumes of 37.5 L·ha^-1^ and 45.0 L·ha^-1^ using the XR110015VS nozzle.

### 3.3 A three-way factorial ANOVA analysis of spray operation parameters on droplet deposition characteristics on the upper canopy of maize plants

The three-way factorial ANOVA revealed distinct mechanistic influences of spray parameters on droplet deposition characteristics in maize upper canopies (**Table 2**). Nozzle type, spray volume, and flight height exhibited significant main effects on all measured metrics: droplet density, coverage rate, and droplet size. For droplet density, significant two-way interactions emerged between nozzle type × flight height and spray volume × flight height, whereas nozzle type × spray volume and the three-way interaction were non-significant. Coverage rate demonstrated greater interactive complexity, with all two-way interactions and the three-way interaction achieving significance. In contrast, droplet size was uniquely dependent on main effects, with no interactive terms contributing to variance.

**Table 2:** Summary of factorial ANOVA for the effect of nozzle types, spray volumes and flight heights on droplet density, coverage rate and droplet size on the upper canopy of maize plant.

Source	DF	Droplet density (droplets·cm^-2^)		Coverage rate (%)		Droplet size (μm)	
		F	*P*	F	*P*	F	*P*
Nozzle type	1	224.2	<0.01	331.8	<0.01	51.8	<0.01
Spray volume	2	58.9	<0.01	56.2	<0.01	8.73	<0.01
Flight height	2	83.1	<0.01	62.6	<0.01	21.4	<0.01
Nozzle type × Spray volume	2	0.48	0.621	7.66	<0.01	2.45	0.101
Nozzle type × Flight height	2	6.59	<0.01	6.37	<0.01	0.36	0.704
Spray volume × Flight height	4	7.45	<0.01	8.89	<0.01	0.75	0.568
Nozzle type × Spray volume × Flight height	4	1.68	0.177	4.73	<0.01	0.51	0.729
Residual	36	–	–	–	–	–	–

### 3.4 Evaluation of control effects against fall armyworm

**Figure 8:**
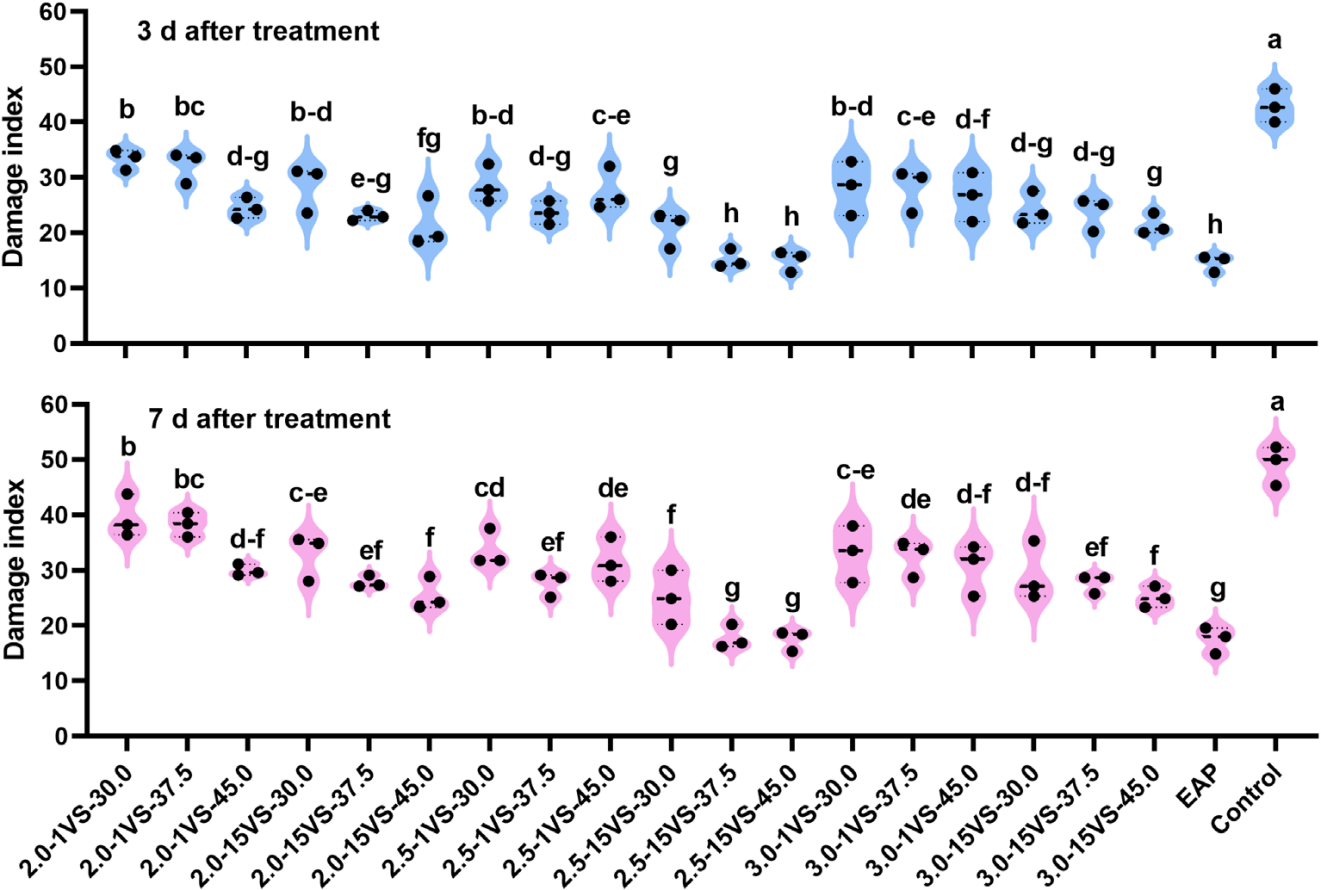
FAW damage index under different treatments in 2019. Different letters on bars indicate significant differences (*P*< 0.05) among treatments.

**Figure 9:**
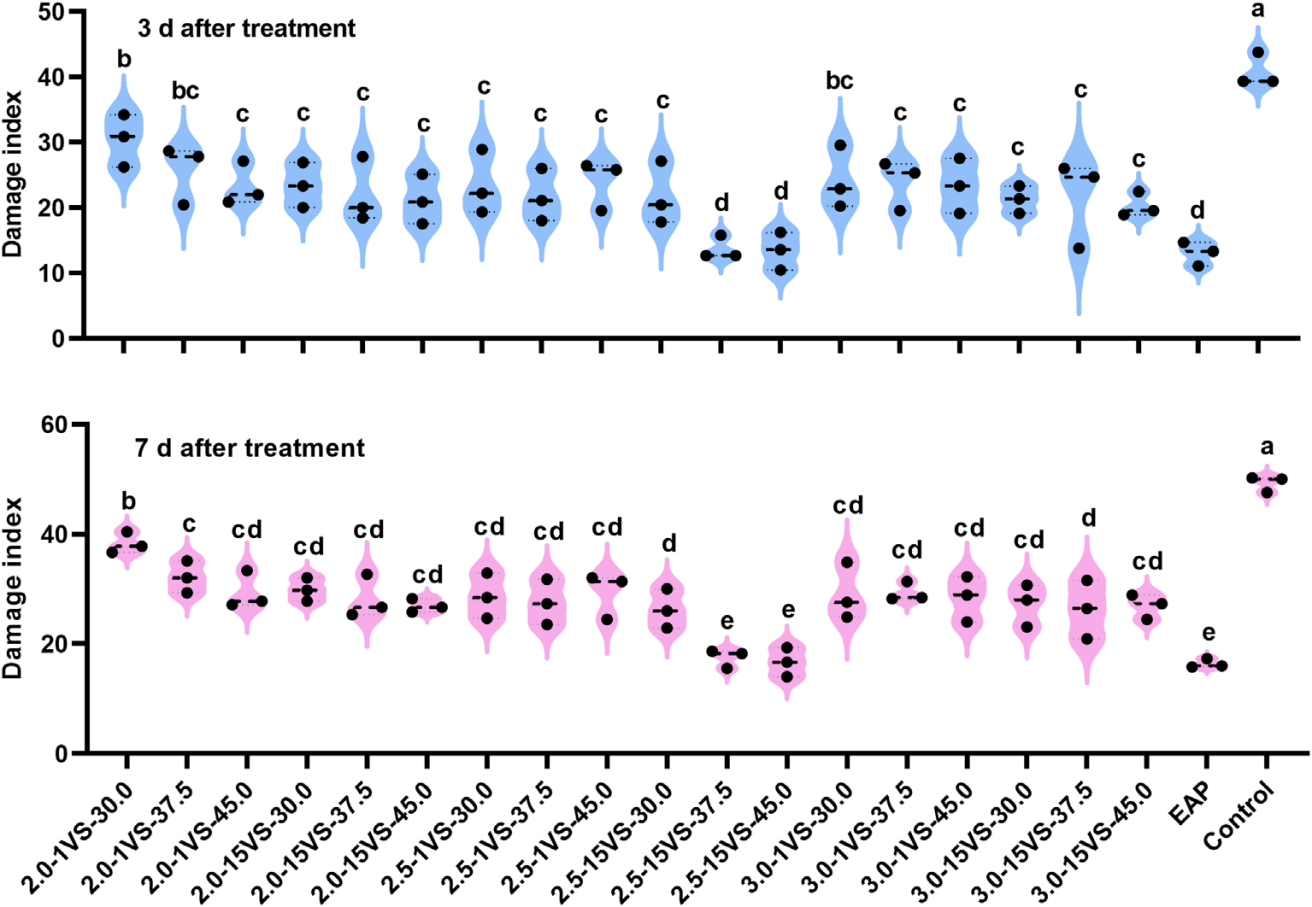
FAW damage index under different treatments in 2020. Different letters on bars indicate significant differences (*P*< 0.05) among treatments.

**Figure 10:**
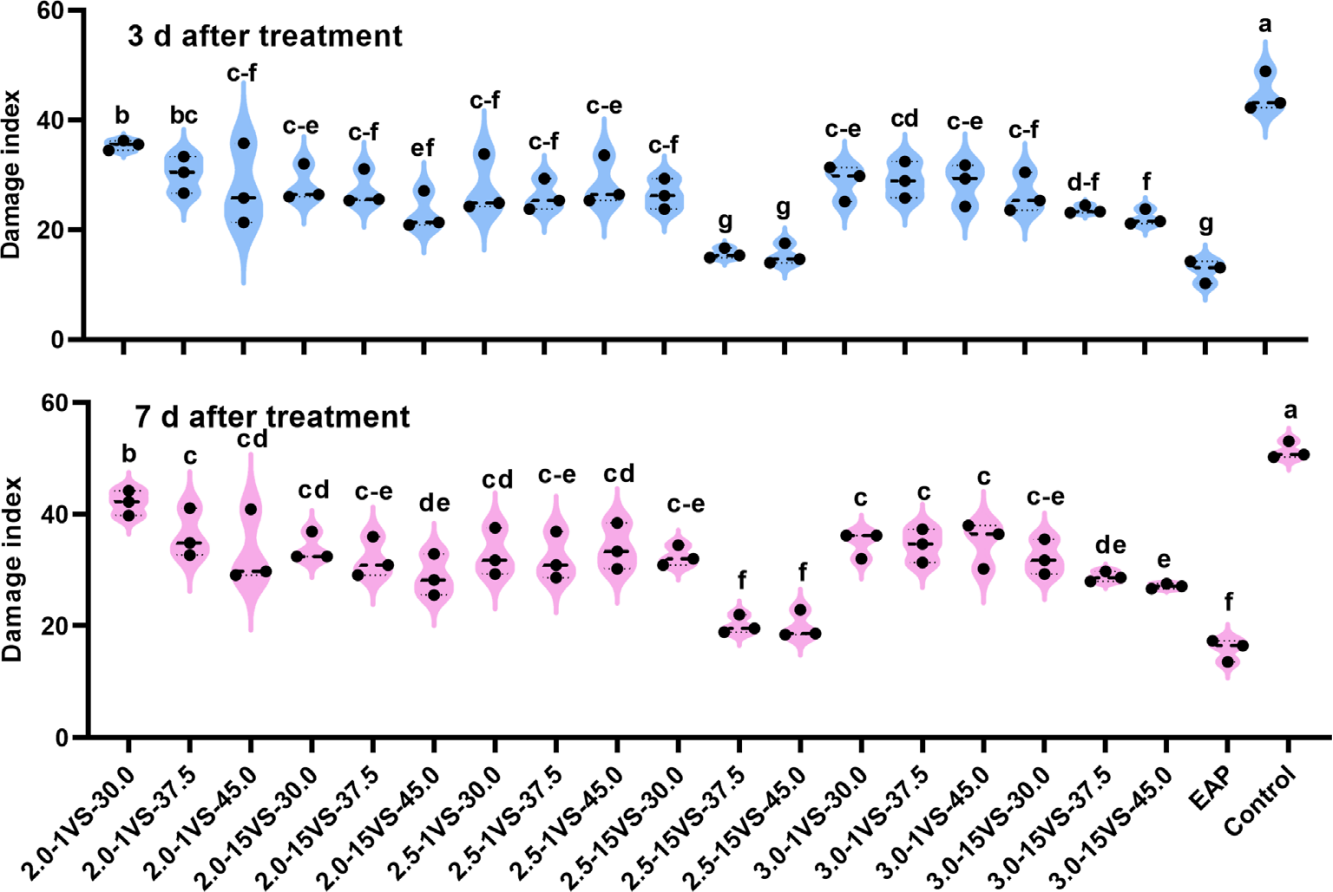
FAW damage index under different treatments in 2021. Different letters on bars indicate significant differences (*P*< 0.05) among treatments.

In 2019, for UAV applications at different flight heights, the Fall Armyworm (FAW) damage indices were consistently lower when using the nozzle XR110015VS compared to the nozzle XR11001VS at the same spray volumes both at 3 days after treatment (DAT) and 7 DAT (**Figure 8**). Additionally, treatments using the XR110015VS nozzle at a flight height of 2.5 meters resulted in lower damage indices than the other two flight height treatments for all three spray volumes. The results confirmed our previous hypothesis: the two optimal spray operation parameter combinations—using a flight height of 2.5 meters with spray volumes of 37.5 L·ha^-1^ or 45.0 L·ha^-1^, and the XR110015VS nozzle—yielded the highest fog density and coverage on the upper canopy of maize and demonstrated the best control effects against FAW. EAP (electric air-pressure knapsack sprayer) application had the lowest damage indices (14.59 and 17.48, respectively) at 3 and 7 DAT. However, these values did not show significant differences compared to treatments with a flight height of 2.5 meters and spray volumes of 37.5 L·ha^-1^ (15.19 and 17.78) or 45.0 L·ha^-1^ (15.04 and 17.48). Similarly, based on FAW damage indices, the control efficacies of EAP application (65.86% and 64.20%, **Supplementary Table S3**) did not differ significantly from the above two UAV treatments (64.63% and 63.90%; 65.02% and 64.51%). Similar results were observed in 2020 and 2021(**Figures 9**, **10**). These findings indicate that optimizing UAV spray operation parameter can achieve comparable FAW control efficacy to traditional equipment. Notably, UAV application technology also offers high efficiency, water savings, and minimizes operator exposure to pesticide contact risks. In summary, farmers are advised to adopt optimized UAV parameters (XR110015VS nozzle, 37.5 or 45.0 L/ha spray volume, 2.5 m flight height) during the whorl stage for effective FAW control.

## 4 Discussion

Fall Armyworm (FAW), a devastating pest of maize, poses significant challenges due to its cryptic larval feeding within maize whorls, which limits insecticide efficacy. Enhancing droplet deposition in the whorl is crucial for improving control efficacy. Unmanned Aerial Vehicles (UAVs), with their downward airflow (wind field), offer advantages by facilitating droplet concentration and penetration into the whorl (Qin et al., 2016). However, varying spray operation parameters such as nozzle type, flight height, and spray volume significantly influence the UAV wind field, thereby affecting droplet deposition on plant canopies (Lou et al., 2018; Chen et al., 2020). This study evaluates these parameters to identify optimal combinations that maximize droplet density and coverage on the upper canopy, where FAW predominantly resides.

Nozzle selection directly affects droplet size distribution, which interacts synergistically with UAV wind field to determine deposition patterns (Fritz and Hoffmann, 2016; Tang et al., 2018). In this study, the XR110015VS nozzle, producing larger droplets, achieved higher droplet density and coverage rate on the upper maize canopy compared to the XR11001VS nozzle. This is consistent with previous findings that larger droplets result in better deposition and coverage on the upper rice canopy (Chen et al., 2020) and on wheat heads (Fritz et al., 2006). Spray volume also plays a key role in droplet deposition. For specific flight heights (2.0 m and 3.0 m), increasing spray volume improved droplet density and coverage on the upper canopy. Similar trends were observed in winter wheat (Shan et al., 2021) and rice (He et al., 2017). However, median spray volumes (37.5 L·ha^-1^) outperformed both lower (30.0 L·ha^-1^) and higher (45.0 L·ha^-1^) volumes at a flight height of 2.5 m, aligning with prior studies (Fritz et al., 2006). Flight height is another critical parameter for achieving ideal droplet deposition. In this study, a height of 2.5 m yielded better results than 2.0 m or 3.0 m, consistent with Tang et al.’s (2018) findings in citrus canopies.

A three-way factorial ANOVA revealed that droplet density and coverage rate are synergistically modulated by parameter interactions, while droplet size remains independent of these effects, reflecting the different physical drivers governing droplet density/coverage rate versus droplet size. Significant interactions among parameters were also observed by Fritz et al. (2006) and Ferguson et al. (2016).

This study demonstrated that droplet deposition distribution varied significantly with different combinations of nozzle type, spray volume, and flight height, which in turn impacted FAW control efficacy. Precise targeting of the upper canopy significantly enhanced the contact rates of insecticides with FAW, thereby improving control efficacy. Optimal treatments using the XR110015VS nozzle at 2.5 m flight height with spray volumes of 37.5 L/ha or 45.0 L/ha achieved superior control effects. These findings align with studies using electric air-pressure knapsack sprayers, where targeting the whorl improved efficacy. Yang et al. (2020) evaluated the control effect of 10% tetrachlorantraniliprole SC by spraying different sites of the maize plant using an EPA sprayer. Their results showed that spraying only the whorl provided better control efficacy than spraying the whole plant with the same dosage. Similarly, Wang Y. et al. (2019) studied the control effect of 20% chlorantraniliprole SC against FAW using the same method and found that spraying half the dose on the maize whorl achieved equivalent control efficacy compared to full-dose application on the whole plant.

While these results are significant for UAV-based FAW control in maize at the whorl stage, maize canopy structure varies across growth stages, influencing droplet deposition patterns. Future work will focus on stage-specific optimization of UAV parameters to ensure consistent FAW suppression while minimizing environmental impact.

## 5 Conclusions

Optimized UAV applications using the XR110015VS nozzle with spray volumes of 37.5 L/ha or 45.0 L/ha at a flight height of 2.5 m achieved FAW control efficacy comparable to traditional knapsack sprayers while reducing water usage by 90%, significantly lowering operational costs and environmental impact, and improving safety for operators. Farmers are advised to adopt these parameters during the whorl stage to effectively control FAW. Future work should focus on adjusting UAV settings to address FAW infestations at different growth stages.

## Data Availability

The original contributions presented in the study are included in the article/**Supplementary Material**. Further inquiries can be directed to the corresponding authors.
