# Supplementary material for: Optimization of spray operation parameters of unmanned aerial vehicle confers adequate levels of control of fall armyworm (Spodoptera frugiperda)
Source: Front Plant Sci. 2025 May 8;16:1581367. doi: 10.3389/fpls.2025.1581367 (PMC12095212; doi:10.3389/fpls.2025.1581367)
Supplement: Supplementary file 1 [file Table1.docx]

**Table S1.** Weather conditions parameters during the test.

| [Treatment](javascript:;) | Spraying  equipment | Flight  height (m) | Nozzle  type | Spray volume  (L·ha^-1^) | | Mean  Temperature (°C) | Mean  Humidity (%) | Mean Wind  Speed (m/s) |
| --- | --- | --- | --- | --- | --- | --- | --- | --- |
| 1 | UAV | 2.0 | XR11001VS | | 30.0 | 24.2 | 54.6 | 0.3 |
| 2 |  |  |  |  | 37.5 | 24.9 | 54.2 | 0.3 |
| 3 |  |  |  |  | 45.0 | 25.7 | 54.0 | 0.3 |
| 4 |  |  | XR110015VS | | 30.0 | 26.5 | 53.2 | 0.4 |
| 5 |  |  |  |  | 37.5 | 27.2 | 53.0 | 0.4 |
| 6 |  |  |  |  | 45.0 | 27.8 | 52.8 | 0.6 |
| 7 |  | 2.5 | XR11001VS | | 30.0 | 28.1 | 52.4 | 0.6 |
| 8 |  |  |  |  | 37.5 | 28.4 | 52.0 | 0.5 |
| 9 |  |  |  |  | 45.0 | 28.9 | 51.2 | 0.7 |
| 10 |  |  | XR110015VS | | 30.0 | 29.2 | 50.9 | 0.8 |
| 11 |  |  |  |  | 37.5 | 29.5 | 50.2 | 1.1 |
| 12 |  |  |  |  | 45.0 | 30.2 | 49.8 | 0.9 |
| 13 |  | 3.0 | XR11001VS | | 30.0 | 30.8 | 49.2 | 0.9 |
| 14 |  |  |  |  | 37.5 | 31.2 | 48.5 | 1.1 |
| 15 |  |  |  |  | 45.0 | 32.2 | 48.1 | 1.2 |
| 16 |  |  | XR110015VS | | 30.0 | 33.4 | 47.8 | 1.4 |
| 17 |  |  |  |  | 37.5 | 34.4 | 47.5 | 1.5 |
| 18 |  |  |  |  | 45.0 | 34.8 | 46.7 | 1.3 |
| 19 | EAP | - | Hollow cone nozzle | | 450.0 | 35.5 | 46.2 | 1.4 |
| 20 | Control | - | - | | - | - | - | - |

**Table S2.** Design and division of the experimental zones.

| **Zone number** | **Treatment code** | | | | | | | | | | | | | | | | | | | |
| --- | --- | --- | --- | --- | --- | --- | --- | --- | --- | --- | --- | --- | --- | --- | --- | --- | --- | --- | --- | --- |
| **A** | **×20** | **×5** | **×4** | **×3** | **×11** | **×6** | **×7** | **×1** | **×2** | **×9** | **×8** | **×10** | **×13** | **×17** | **×15** | **×14** | **×12** | **×19** | **×18** | **×16** |
| **B** | **×10** | **×12** | **×16** | **×15** | **×13** | **×17** | **×8** | **×19** | **×5** | **×4** | **×7** | **×9** | **×2** | **×3** | **×18** | **×11** | **×14** | **×1** | **×6** | **×20** |
| **C** | **×20** | **×3** | **×1** | **×19** | **×4** | **×2** | **×18** | **×10** | **×17** | **×7** | **×13** | **×15** | **×6** | **×5** | **×14** | **×16** | **×12** | **×9** | **×8** | **×11** |

**Table S3** Damage index and relative control eﬃcacy of FAW evaluated at 3 and 7 days after treatment (DAT) in **2019**. Means (SE) with different letters in each column indicate statistically difference (significant level α = 0.05). Relative control efficacy (%) = [(D_C_- D_T_)/D_T_] × 100%, D_C_ is the damage index in treatment plot and D_T_ is the damage index in control plot.

| **Treatment** | **3 DAT** | | **7 DAT** | |
| --- | --- | --- | --- | --- |
|  | **Damage index** | **Control eﬃcacy (%)** | **Damage index** | **Control eﬃcacy (%)** |
| 1 | 33.33 (1.05) b | 22.22 (1.00) e | 39.48 (2.21) b | 19.65 (3.73) e |
| 2 | 32.15 (1.63) bc | 25.08 (1.92) de | 38.30 (1.29) bc | 22.02 (2.22) de |
| 3 | 24.44 (1.10) defg | 42.95 (2.16) bc | 29.93 (0.61) def | 39.03 (1.63) bc |
| 4 | 28.44 (2.45) bcd | 33.05 (8.05) cde | 32.81 (2.42) cde | 32.72 (7.27) cde |
| 5 | 23.04 (0.52) efg | 46.07 (2.82) bc | 27.85 (0.63) ef | 43.07 (3.69) bc |
| 6 | 21.48 (2.61) fg | 50.16 (4.32) b | 25.48 (1.72) f | 48.19 (2.63) b |
| 7 | 28.67 (1.98) bcd | 32.70 (6.90) cde | 33.70 (1.93) cd | 30.91 (6.93) cde |
| 8 | 23.63 (1.22) defg | 44.85 (2.46) bc | 27.63 (1.27) ef | 43.60 (3.77) bc |
| 9 | 27.56 (2.26) cde | 35.95 (2.76) cd | 31.63 (2.34) de | 35.84 (2.39) bcd |
| 10 | 20.81 (1.87) g | 50.98 (6.13) b | 25.04 (2.82) f | 48.44 (7.98) b |
| 11 | 15.19 (0.97) h | 64.63 (1.32) a | 17.78 (1.24) g | 63.90 (1.44) a |
| 12 | 15.04 (1.09) h | 65.02 (1.42) a | 17.48 (1.08) g | 64.51 (0.89) a |
| 13 | 28.22 (2.83) bcd | 33.77 (8.33) cde | 33.11 (2.96) cde | 32.12 (8.36) cde |
| 14 | 28.07 (2.27) cde | 34.38 (5.74) cde | 32.44 (1.92) de | 33.67 (5.73) cde |
| 15 | 26.59 (2.57) def | 37.52 (7.48) bcd | 30.52 (2.67) def | 37.48 (7.03) bc |
| 16 | 24.22 (1.73) defg | 43.25 (5.04) bc | 29.26 (3.08) def | 40.34 (6.40) bc |
| 17 | 23.70 (1.75) defg | 44.60 (4.45) bc | 27.70 (0.96) ef | 43.44 (3.47) bc |
| 18 | 21.41 (1.09) g | 49.88 (3.47) b | 25.11 (1.10) f | 48.73 (3.30) b |
| 19 | 14.59 (0.85) h | 65.86 (2.54) a | 17.48 (1.37) g | 64.20 (3.91) a |
| 20 | 42.89 (1.74) a | - | 49.19 (2.03) a | - |

**Table S4** Damage index and relative control eﬃcacy of FAW evaluated at 3 and 7 days after treatment (DAT) in **2020**. Means (SE) with different letters in each column indicate statistically difference (significant level α = 0.05). Relative control efficacy (%) = [(D_C_- D_T_)/D_T_] × 100%, D_C_ is the damage index in treatment plot and D_T_ is the damage index in control plot.

| **Treatment** | **3 DAT** | | **7 DAT** | |
| --- | --- | --- | --- | --- |
|  | **Damage index** | **Control eﬃcacy (%)** | **Damage index** | **Control eﬃcacy (%)** |
| 1 | 30.44 (2.32) b | 25.54 (3.90) c | 38.30 (1.12) b | 22.26 (1.67) d |
| 2 | 25.63 (2.61) bc | 37.30 (5.56) bc | 32.15 (1.67) c | 34.79 (2.58) c |
| 3 | 23.33 (1.92) c | 42.57 (5.81) b | 29.41 (1.97) cd | 40.13 (5.12) bc |
| 4 | 23.41 (1.99) c | 42.49 (5.48) b | 29.85 (1.22) cd | 39.28 (3.50) bc |
| 5 | 22.07 (2.89) c | 46.27 (4.99) b | 28.22 (2.26) cd | 42.77 (4.05) bc |
| 6 | 21.19 (2.19) c | 47.65 (6.87) b | 26.89 (0.71) cd | 45.39 (1.53) bc |
| 7 | 23.48 (2.83) c | 41.96 (8.49) b | 28.67 (2.38) cd | 41.79 (4.73) bc |
| 8 | 21.70 (2.33) c | 47.06 (3.95) b | 27.56 (2.38) cd | 44.02 (4.86) bc |
| 9 | 23.93 (2.19) c | 41.44 (4.66) bc | 29.26 (2.42) cd | 40.48 (5.45) bc |
| 10 | 21.78 (2.78) c | 46.39 (7.67) b | 26.30 (2.06) d | 46.71 (3.41) b |
| 11 | 13.70 (1.04) d | 66.52 (1.28) a | 17.48 (0.97) e | 64.56 (1.40) a |
| 12 | 13.41 (1.67) d | 67.31 (3.16) a | 16.67 (1.54) e | 66.24 (2.68) a |
| 13 | 24.22 (2.78) bc | 40.38 (7.77) bc | 29.11 (2.99) cd | 41.03 (5.31) bc |
| 14 | 23.85 (2.18) c | 41.54 (5.23) bc | 29.33 (1.00) cd | 40.34 (3.11) bc |
| 15 | 23.33 (2.44) c | 42.68 (6.52) b | 28.37 (2.39) cd | 42.23 (5.76) bc |
| 16 | 21.26 (1.22) c | 47.59 (4.62) b | 27.26 (2.21) cd | 44.61 (4.63) bc |
| 17 | 21.48 (3.87) c | 47.62 (8.73) b | 26.30 (3.08) d | 46.77 (5.55) b |
| 18 | 20.30 (1.09) c | 50.33 (0.94) b | 26.89 (1.30) cd | 45.47 (1.77) bc |
| 19 | 13.04 (1.04) d | 67.81 (3.54) a | 16.37 (0.49) e | 66.77 (0.73) a |
| 20 | 40.81 (1.48) a | - | 49.26 (0.85) a | - |

**Table S5** Damage index and relative control eﬃcacy of FAW evaluated at 3 and 7 days after treatment (DAT) in **2021**. Means (SE) with different letters in each column indicate statistically difference (significant level α = 0.05). Relative control efficacy (%) = [(D_C_- D_T_)/D_T_] × 100%, D_C_ is the damage index in treatment plot and D_T_ is the damage index in control plot.

| **Treatment** | **3 DAT** | | **7 DAT** | |
| --- | --- | --- | --- | --- |
|  | **Damage index** | **Control eﬃcacy (%)** | **Damage index** | **Control eﬃcacy (%)** |
| 1 | 35.41 (0.52) b | 20.49 (3.81) f | 42.07 (1.29) b | 18.01 (2.64) e |
| 2 | 30.15 (1.93) bc | 32.58 (2.11) e | 36.22 (2.53) c | 29.55 (3.77) d |
| 3 | 27.63 (4.27) cdef | 37.79 (10.40) cde | 33.26 (3.82) cd | 35.02 (8.23) cd |
| 4 | 28.15 (1.93) cde | 36.54 (6.51) de | 33.93 (1.48) cd | 33.83 (3.47) cd |
| 5 | 27.33 (1.89) cdef | 38.92 (1.41) bcde | 32.00 (2.06) cde | 37.76 (2.90) bcd |
| 6 | 23.11 (2.00) ef | 48.39 (2.05) bc | 28.89 (2.14) de | 43.83 (3.20) bc |
| 7 | 27.63 (3.08) cdef | 38.49 (3.79) bcde | 32.89 (2.44) cd | 36.04 (3.72) cd |
| 8 | 26.15 (1.65) cdef | 41.16 (4.81) bcde | 32.15 (2.46) cde | 37.27 (5.38) bcd |
| 9 | 28.44 (2.58) cde | 36.57 (2.62) de | 34.00 (2.40) cd | 33.86 (3.68) cd |
| 10 | 26.44 (1.61) cdef | 40.84 (1.27) bcde | 32.44 (1.05) cde | 36.82 (1.16) cd |
| 11 | 15.63 (0.53) g | 64.68 (2.74) a | 20.15 (0.95) f | 60.69 (2.27) a |
| 12 | 15.41 (1.09) g | 65.13 (3.49) a | 20.00 (1.45) f | 60.96 (3.29) a |
| 13 | 28.74 (1.87) cde | 35.63 (3.61) de | 34.81 (1.41) c | 32.20 (2.25) d |
| 14 | 29.04 (1.93) cd | 35.06 (2.66) e | 34.44 (1.74) c | 32.97 (2.38) d |
| 15 | 28.44 (2.23) cde | 36.12 (5.89) de | 34.89 (2.38) c | 32.07 (4.29) d |
| 16 | 26.44 (2.06) cdef | 40.92 (2.29) bcde | 32.22 (1.81) cde | 37.31 (2.46) bcd |
| 17 | 23.63 (0.41) def | 46.93 (1.56) bcd | 28.81 (0.52) de | 43.82 (1.60) bc |
| 18 | 22.15 (0.82) f | 50.09 (3.66) b | 27.11 (0.26) e | 47.17 (0.49) b |
| 19 | 12.52 (1.19) g | 71.87 (2.32) a | 15.78 (1.14) f | 69.31 (1.85) a |
| 20 | 44.74 (2.09) a | - | 51.33 (0.90) a | - |
